# Rumination and interoceptive accuracy predict the occurrence of the thermal grill illusion of pain

**DOI:** 10.1186/2050-7283-2-22

**Published:** 2014-07-18

**Authors:** Raymonde Scheuren, Stefan Sütterlin, Fernand Anton

**Affiliations:** Institute of Health and Behaviour, Integrative Research Unit on Social and Individual Development, University of Luxembourg, Luxembourg, Grand-Duchy of Luxembourg; Section of Psychology, Lillehammer University College, Lillehammer, Norway; Research Group Health Psychology, University of Leuven, Leuven, Belgium; Department of Psychosomatic Medicine, Division of Surgery and Clinical Neuroscience, Oslo University Hospital – Rikshospitalet, Oslo, Norway

## Abstract

**Background:**

While the neurophysiological mechanisms underlying the thermal grill illusion of pain (TGI) have been thoroughly studied, psychological determinants largely remain unknown. The present study aimed to investigate whether cognitive and affective personality traits encompassing rumination, interoception, and suggestibility may be identified as characteristics favouring the elicitation of paradoxical pain experiences.

**Methods:**

The dominant hand of 54 healthy volunteers was stimulated with a water-bath driven thermal grill providing an interlaced temperature combination of 15 and 41°C. Pain intensity and pain unpleasantness perceptions were rated on a combined verbal-numerical scale (NRS). Traits were assessed via questionnaires, the heartbeat-tracking task, and warmth suggestions.

**Results:**

Logistic regression analyses uncovered trait rumination and interoceptive accuracy (IA) as major predictors of the likelihood of the illusive pain occurrence (all *p* < .05). Rumination and suggestibility had an impact on unpleasant pain perceptions.

**Conclusion:**

Our findings allowed identifying psychological factors substantially involved in the individual pre-disposition to reporting painful sensations in the thermal grill paradigm. These psychological characteristics may also be relevant in the context of central neuropathic pain, which to a large extent incorporates the same neural pathways.

## Background

### Thermal grill illusion of pain

Since Thunberg revealed in 1896 that interlaced and non-noxious cold and warm stimuli applied to the skin generate the thermal grill illusion of pain (TGI), a paradoxical feeling of pain, the underlying neurophysiological mechanisms have thoroughly been studied (Craig and Bushnell 1994; Craig et al. 1996, 2000; Kern et al. 2008; Lindstedt et al. 2011b). Functional imaging studies on the TGI have uncovered an involvement of cerebral structures like the contralateral thalamus (Lindstedt et al. 2011b), the anterior cingulate cortex (Craig et al. 1996), and the insula (Craig et al. 2000) that are to a large extent also involved in the regulation of emotions and of interoceptive awareness (Craig 2002). Since the identified neuroanatomical substrates suggest that the illusive pain might share common mechanisms with central neuropathic pain, the thermal grill has been used as a model for the investigation of central pain-related neural activity (Craig 2008).

### Inter-individual differences in thermal grill responsiveness

A number of studies have provided evidence for inter-individual differences in thermal grill-related pain sensitivity (Boettger et al. 2011; Bouhassira et al. 2005, Lindstedt et al. 2011a). It could be shown that painful sensations in response to thermal grill stimulation were only perceived by about one third of the participants. Those individuals were qualified as responders to the TGI, whereas those who reported non-painful warm or/and cold sensations or very low pain were described as non- or poor-responders (Boettger et al. 2013; Bouhassira et al. 2005). The reasons for the observed inter-individual differences in TGI susceptibility remain unknown to this point.

We hypothesized that the described differences in susceptibility to the expression of pain could at least partly be related to psychological features. The identification of the previously mentioned cortical areas involved in the TGI as well as in emotional regulation (Craig 2002) seems to underpin this assumption. Further support may be derived from the multidimensional character of pain (Wiech and Tracey 2009) implying that psychological factors are heavily involved in the regulation of pain sensitivity in different pain conditions or experimental pain models. It could in particular be shown that affective and cognitive characteristics promote discrepancies between induced and perceived pain intensity levels (Pennebaker 1999; Wiech and Tracey 2009). Subjects with high levels of anxiety or attention to pain did e.g. display more pronounced ratings to noxious stimulation than people exhibiting lower values of the mentioned psychological characteristics (Tang and Gibson 2005).

So far however, investigations on the impact of psychological features on the manifestation of paradoxical pain responses remain very scarce. Only the pain enhancing effects of depression and sad mood on thermal grill-activated central pain processing have been confirmed in clinical studies (Boettger et al. 2011; Piñerua-Shuhaibar et al. 2011).

### Personality traits and pain

In this framework, we turned towards personality traits that have been identified as important pain modulating factors in classical pain research (i.e. under conditions of evident noxious stimulation). Psychological characteristics such as pessimism, pain catastrophizing, anxiety and related negative affectivity (Crombez et al. 1998; Sullivan et al. 2001a; Affleck et al. 2001), maladaptive coping styles (Keefe et al. 1989; Smith and Alloy, 2009) or biased cognitive processes (Crombez et al. 2013) have repeatedly been described to be associated with increased pain perceptions or pain distortions (Crombez et al. 1998; Edwards et al. 2006; James and Hardardottir 2002; Sullivan et al. 2001a, 2005; Tang and Gibson, 2005; Wiech and Tracey, 2009).

#### Trait pessimism versus trait optimism

Experimental (Affleck et al. 2001, Geers et al. 2008; Mahler and Kulik 2000) and clinical (Goodin et al. 2013) findings suggest that pessimistic individuals feel more pain than optimistic pain patients or healthy volunteers. It has been claimed that pessimistic persons turn more attention to pain, have negative expectations concerning future outcomes, are rather convinced of their inability to deal with problems, and refer to maladaptive coping methods (Geers et al. 2008). Optimists in contrast are more likely to expect favorable outcomes and relate to positive cognitions and behaviours to adjust to or disengage from negative or painful experiences (i.e. approach coping style; Goodin et al. 2013). Hanssen et al. (2013) have shown that the relationship between optimism and low pain intensity ratings is mediated by low pain catastrophizing.

#### Trait pain catastrophizing, trait anxiety, and trait rumination

It has been observed that high trait pain catastrophizing is concomitant with increased anxiety, attention to and anticipation of pain and enhances painful sensations (Crombez et al. 1998; Edwards et al. 2006; Keefe et al. 1989; Sullivan et al. 2001a, 2005, Van Damme et al. 2004). There also exists a relationship between high trait anxiety and increased pain intensity resp. state anxiety (Ploghaus et al. 2001; Tang and Gibson 2005). The inability to repress pain-related feelings and thoughts constitutes a major stressor for catastrophizing and anxious persons and strongly promotes ruminative thinking (Edwards et al. 2006). Trait rumination is characterized by perseverative thinking on negative events and a deficient cognitive control of ongoing thoughts and is considered as a dimension of the pain catastrophizing construct [cf. Pain Catastrophizing Scale (PCS), Sullivan et al. 1995]. In high ruminators, goal-directed and problem-based coping is hampered by adverse expectations and difficulties in accepting upsetting episodes or in deflecting their attention from problems and bad feelings (Smith and Alloy 2009).

#### Expectations and suggestibility

Pain magnitude and pain unpleasantness have been reported to depend on the intensity of expected pain (Atlas and Wagner 2012; Boersma and Linton 2006; Tracey 2010). In placebo-related settings, low expectations have been found to play a pain-reducing role (Price et al. 1999), whereas high pain expectancy promoted a negative response or nocebo effect while being interrelated with more anxiety and worrisome feelings (Benedetti et al. 2007; Sawamoto et al. 2000). Another psychological characteristic closely linked to positive and negative pain-related placebo effects is suggestibility (De Pascalis et al. 2002; Staats et al. 1998). It is widely accepted that pain may be lowered in highly suggestible participants following a suggestion of an efficient pain-relieving drug (De Pascalis et al. 2002) or be increased following nocebo stipulations (Staats et al. 1998).

#### Interoceptive accuracy

The psychophysiological feature interoceptive accuracy (IA) was considered as an additional potential predictor of pain responses to the thermal grill application. The ability to discern internal bodily states is regarded as a stable trait (Tsakiris et al. 2011) and has been highly associated with a tendency of experiencing more intense emotions (Wiens et al. 2000) and of being inclined to more anxiety and catastrophizing (Critchley et al. 2004; Pollatos et al. 2007). This proneness to stronger emotional feelings can lead to a dysfunctional cognitive processing of interoceptive states and to a misjudgement of bodily signals (Wölk et al. 2013). As a consequence, the experience of somatic symptoms is enhanced (Critchley et al. 2004) or over-reported (Barsky and Borus 1999). Biased emotional decision-making (Garfinkel and Critchley 2013; Sütterlin et al. 2013; Wölk, et al. 2013) and an expectation of possibly negative consequences have also been shown in individuals scoring high in interoceptive accuracy. Interestingly, in research based on suprathreshold noxious stimulation, Pollatos et al. (2012) revealed that participants correctly perceiving their cardiac signals had lower pain threshold and tolerance levels than interoceptively less accurate individuals. Wiech and Tracey (2009) reported that interoception is linked to higher pain perceptions when negative emotional factors like anxiety, catastrophizing, and expectation of pain are involved.

The relationships between pain-related emotional and cognitive personality traits and pain perceptions described in the present study have been derived from classical pain research where they explain inter-individual differences in pain responsiveness to noxious experimental stimulation or to pathological pain conditions. We hypothesized that these psychological and psychophysiological features might not only be involved in the quantitative modulation of pain responsiveness, but also in the qualitative crossover from non-painful to painful sensations in the absence of peripheral noxious input. An identification of dispositional feelings and thoughts affecting thermal grill perceptions was expected to improve the understanding of differential paradoxical pain sensitivity and potentially to provide additional insight into the processes influencing central neuropathic pain syndromes. To test our hypothesis, we first identified responders and non-responders to the thermal grill stimulation by means of subjective ratings of thermal grill-related pain intensity and pain unpleasantness (Boettger et al. 2011, 2013; Bouhassira et al. 2005). In a further step, the personality features trait pessimism–optimism, trait pain catastrophizing, trait anxiety, trait rumination, expectancy of pain, suggestibility, and IA were individually assessed in the participants to characterize responders and non-responders to the TGI and to provide evidence by means of logistic regression analyses that volunteers displaying high levels of specific pain-related traits are more likely to feel the TGI.

## Methods

### Participants

A sample of 66 healthy participants comprising student and staff populations of the University of Luxembourg was screened. Health-related issues were retrieved with a medical history questionnaire. Depression or mood problems were in addition appraised on the basis of the self-report trait and state questionnaires. Only volunteers without psychological-, cardiovascular-, neurological-, pain-, and skin-related disorders or problems were included in the study. Drugs and pain medication intake 24 hours before experimental testing were also considered as exclusion criteria. Prior to the experimental session, participants were informed that the study was about investigating potential differences in temperature-related perceptions. Furthermore, the volunteers were briefed about the anonymization of the obtained data and their right of withdrawal without any further consequences. All participating volunteers gave informed consent. The true scientific rationale of the study was provided in the debriefing at the end of the laboratory session. The experimental protocol was approved by the National Research Ethics Committee (ref. 1102–59) and complied with the ethical guidelines of the International Association for the Study of Pain (IASP; Charlton, 1995). Ten participants were excluded from the study since they experienced pain in the control conditions i.e. when stimulated with neutral 32°C (normal skin temperature) in combination with either the warm or cold temperature used for the elicitation of the TGI. The 11^th^ ‘outlier’ could not be included in the final sample due to technical problems with the thermal grill and incomplete pain ratings. The data of one participant displaying depressive symptoms were excluded from the analyses. The final sample included 54 participants [26 males, 28 females, M = 24.1 years (SD = 6.01), range 18–51 years]. All volunteers were financially compensated.

### Material

#### Thermal grill and accessories

**Figure 1:**
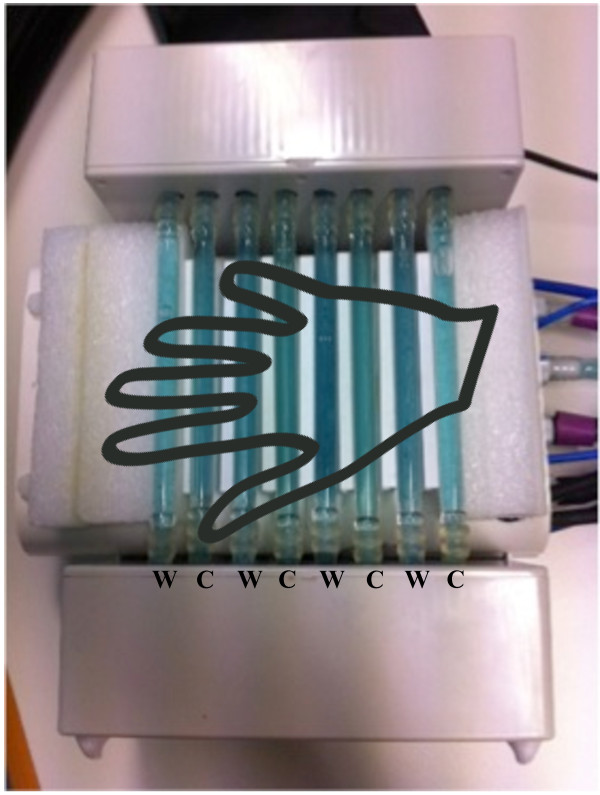
**Custom-** **built thermal grill device.** W: warm tubes; C: cold tubes.

A custom-built and water-bath driven thermal grill device was used to elicit the paradoxical pain (Curio, I., PhD, Medical Electronics, Bonn/Germany). The thermal grill was composed of eight alternating cold and warm pipes made of borosilicate glass. The glass pipes were spaced at a distance of 7.5 mm by means of separating bars to prevent any ‘mixing phenomenon’ between pipes. The bars were made of 5 mm hollow (thickness 0.5 mm) polyvinyl chloride (PVC) with negligible thermal conductivity. The total surface of the rectangular pipes measured 20 × 10 cm (see Figure 1). The temperatures were regulated with two separate thermoelectric recirculating chillers (T255P, ThermoTek Inc.) delivering the water to the grill pipes through separate flexible and insulated plastic conduits. The flow rate of the pump was 3,86 l/min, approx. 15 ml/s per glass pipe. The volume of one glass pipe was about 16.5 cm^3^. The fluid content of each pipe was exchanged at a rate of about one second. The fluid temperature was continuously controlled with a digital thermometer (PL-120 T2, Voltcraft; visual display of T1-T2 temperatures in °C) placed at the manifold, where the water flow was distributed to the glass pipes. Previous measurements have shown that a stationary temperature distribution was reached about 3 s after applying the skin to the pipes.

**Figure 2:**
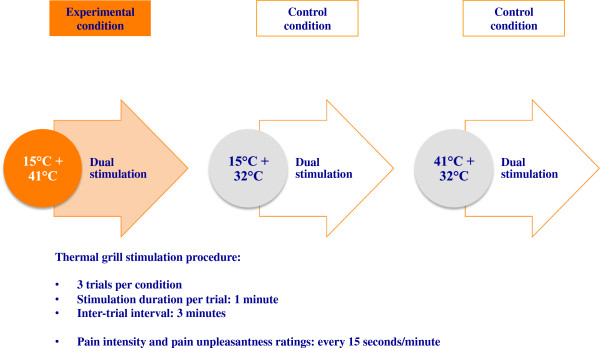
**Thermal grill stimulation sequences.**

For the experimental thermal grill condition, we preferred stimulating all participants with the same fixed temperature combination of 15°C and 41°C, instead of individualized temperatures defined in association with previously assessed thermal pain thresholds (as described in studies using Peltier-driven thermal grills; Bouhassira et al. 2005). This choice was based on the circumstance that water-bath-related temperature changes are time-consuming and on the finding that larger differences between cold and warm grill temperatures allow generating reasonable pain intensities (Boettger et al. 2011; Bouhassira et al. 2005; Lindstedt et al. 2011a). The chosen temperature combination of 15°C and 41°C (difference of 26°C degrees; Boettger et al. 2011; Bouhassira et al. 2005; Lindstedt et al. 2011a) was applied throughout the one-minute trials of the experimental condition (see Figure 2). An inter-stimulus-interval (ISI) of three minutes was always respected between the trials. The same temporal procedure was applied in the two subsequent control conditions. In control condition 1, the cold temperature of 15°C was combined with the baseline temperature of 32°C, whereas in control condition 2 the warm temperature of 41°C was set together with the 32°C input (see Figure 2). As an alternative to previous research procedures using single stimulations (e.g. 15°C in all thermal grill tubes) for control, we preferred providing dual interlaced temperature stimulations mimicking the spacing of the respective temperatures in the experimental 15°C/41°C phase. The order of the stimulation conditions was not counterbalanced to allow for comparability between the responder and non-responder groups.

The thermal grill stimuli were always applied at the palmar side of the dominant hand. The hand of the participant was placed on the thermal stimulation surface and held in place with a cuff to warrant an equilibrated and integral contact between the hand and the grill bars. The cuff was inflated with a sphygmomanometer (mmHg) until a gentle pressure held the hand in the adequate position. The contact area of the skin to the glass bars (effective surface) was approximately 0.8 cm × 8 (effective glass pipe width in contact with skin × 8 pipes) × 11 cm (width of the hand) = 70.4 cm^2^. Applying a pressure of 0.7 MPa (0.071 kp/cm^2^ = 50 mmHg), the resulting force was about 0.5 kp. It was considered quite unlikely that the gentle pressure applied with the cuff continuously stimulated the cutaneous mechanoreceptors (which adapt fairly quickly) and influenced the perception of the TGI or changed the suggestibility of the participants. Furthermore, although a modulation of spinal nociceptive processing by concomitant low threshold A-fiber input is well established (Handwerker et al. 1975), this effect was not expected to play a role in the present stimulus conditions, which do not involve any nociceptive input to the dorsal horn that could be modulated. After each stimulation phase, the cuff was detached and the volunteers removed the hand from the grill during the ISI to prevent carry-over effects (Boettger et al. 2011; Bouhassira et al. 2005). Between the different stimulation conditions, a time interval of about 10 minutes had to be respected to allow for adjustment of the targeted grill temperature combination.

#### Contact heat stimulator

During the so-called generalization suggestion of the Warmth Suggestibility Scale (WSS; Gheorghiu et al. 2003), thermal stimuli of a baseline temperature of 32°C (Morin and Bushnell 1998; Lindstedt et al. 2011a) were applied with a Peltier-driven and temperature controlled contact heat evoked potential (CHEP) stimulator (Pathway, Cheps, Medoc Ltd, Israel) and a thermode with a contact surface of 30x30 mm. Constant warm stimuli of one minute duration were delivered to the non-dominant hand of the participant.

#### Physiological assessments

The MP150 Data Acquisition System (BIOPAC Systems Inc., USA) was used to record the cardiac activity during the heartbeat-tracking task. Disposable pre-gelled Ag-AgCl electrodes (diameter 35 mm, EL502, Biopac Systems) were placed below the right clavicle and below the left lower rib to perform the standard precordial lead II electrocardiogram (ECG; ECG100C; 0.5 Hz high pass filtering, R-wave output mode, signal gain 500). Subjects were grounded through a similar electrode positioned below the right lower rib. ECG recordings were continuously computed during the heart rate perception measure. Physiological data collection and offline analyses of the frequency of the recorded R-waves were realized with the AcqKnowledge Software package (BIOPAC Systems Inc., USA).

### Measures

#### Pain rating scales

*Expectancy of pain* was assessed with a visual analogue scale (VAS) measuring 100 mm. The scale was anchored from 0 = *no pain expected* to 100 = *intolerable pain expected*. The intensity of pain participants had expected to feel during the experiment before coming to the lab was assessed at the end of the experimental session to avoid the occurrence of undesirable pain suggestions potentially having an impact on the responses to the subsequently presented sensory stimuli (Arntz and Claassens 2004; Wiech et al. 2008).

*Pain intensity and pain unpleasantness ratings*. The affective-motivational component of pain was assessed in addition to the sensory-discriminative aspect since both dimensions can vary independently in the sense that emotional characteristics may affect pain unpleasantness sensations without however changing the sensory pain component (Villemure and Bushnell 2002). Unpleasantness is moreover often increased in response to the thermal grill stimulation (Bouhassira et al. 2005; Lindstedt et al. 2011a). The subjective evaluation of the intensity and unpleasantness of the thermal grill-induced sensations was done with a combined verbal-numerical rating scale (NRS; Gracely 2006; Lindstedt et al. 2011a) involving a continuous range from 0–100 and a set of verbal descriptors of the various scale increments. The 0 – < 20 range was used for the indication of no or non-painful thermal sensations [0 = *no sensation*; 10 = *warm*/*cold*; 20 = *grill pain threshold* (GPT)]. The ≥ 20–100 range was used for the assessment of the painful perceptions [20 = *grill pain threshold* (GPT); 30 = *very weak pain*/*unpleasantness*; 40 = *weak pain*/*unpleasantness*; 50 = *moderate pain*/*unpleasantness*; 60 = *slightly strong pain*/*unpleasantness*; 70 = *strong pain*/*unpleasantness*; 80 = *very strong pain*/*unpleasantness*; 90 = *nearly intolerable pain*/*unpleasantness*; 100 = *intolerable pain*/*unpleasantness*)]. It may be emphasized that the described subdivision implies that a pain rating of 20-NRS on our scale corresponds to a rating of 0-NRS (=*no pain*) on an ordinary scale, a 30-NRS rating is equivalent to 10-NRS (=*very weak pain*/*unpleasantness*), etc. The participants were explicitly instructed that the first part of the scale ranging from 0 to < 20-NRS-values should be used for the indication of non-existent or non-painful thermal sensations, whereas values ≥ 20 would always quantify intensity or unpleasantness levels related to the perception of pain. For the accurate assessment of their perceptions, the volunteers were allowed to use increments of 1.0 or 0.5 decimals on the NRS. They were furthermore instructed to rate the sensory-discriminative component of pain before the affective-motivational pain dimension. Pain ratings were orally delivered in intervals of 15 seconds during each thermal grill stimulation period (i.e. four sensory and four affective pain ratings per one-minute stimulation trial, three trials per condition; see Figure 2) since the dominant hand of the participants was positioned on the grill.

#### Self-report questionnaires

*State*- *and trait anxiety*. Inter-individual differences in state and trait anxiety were assessed with the Form Y of the State-Trait Anxiety Inventory (STAI; Spielberger et al. 1983). The questionnaire is based on 40 items and a 4-point Likert scale ranging from 1 = *not at all* to 4 = *very much so*. The first 20 expressions involve the state anxiety items, whereas trait anxiety is assessed with the statements numbered 21 – 40. Internal consistency (α = .95 and .93; Grös et al. 2007) and reliability of the STAI scales (Cronbach’s α of .93; Balsamo et al. 2013) have been reported to be high.

*Trait pain catastrophizing* was assessed via the Pain Catastrophizing Scale (PCS; Sullivan et al. 1995). On the basis of a 5-point scale (0 = *not at all* to 4 = *all the time*), the items of the rumination, magnification, and helplessness subscales of the PCS are related to feelings and thoughts associated with painful experiences of the past. Higher catastrophizing values (possible range 0–52) indicate greater emotional reactions to painful stimuli. The PCS has been classified as instrument with adequate to excellent internal consistency [coefficient alpha of total PCS: .87; rumination: .88; magnification: .66; helplessness: .78 (Sullivan et al. 1995)].

*Dispositional Pessimism*/*Optimism*. The revised version of the Life Orientation Test (LOT-R; Scheier et al. 1994) was used for the measurement of trait pessimism versus trait optimism in the participants (Herzberg et al. 2006). High scores indicate optimism and positive expectations for the future. The good validity and reliability of the LOT-R questionnaire have repeatedly been confirmed (Herzberg et al. 2006; Scheier et al. 1994).

The magnitude of *trait rumination* was determined with a short version of the Response Style Questionnaire (RSQ; Nolen-Hoeksema and Morrow 1991; Sütterlin et al. 2012). The 10 items refer to the subscales brooding (i.e. thoughtful contemplation of own problems and feelings of distress associated with negative mood and low or inexistent problem-solving behaviour) and reflection (i.e. inward-directed analysis of depressed feelings and potential engagement in adaptive actions) (Treynor et al. 2003). The self-report scores range from 0 = *never* to 3 = *always* and are summed as overall score reaching values between 0 and 30.

#### Interoceptive accuracy

IA was assessed with the heartbeat-tracking task (Herbert et al. 2012; Pollatos et al. 2007; Schandry 1981). Participants were asked to mentally count the number of heartbeats they felt during the time intervals of 25, 35, and 45 seconds. The experimenter orally informed the volunteers of the beginning and the end of the different time intervals. A pause of 60 seconds was implemented between all time periods. The participants were not allowed to use any additional help or strategies (e.g. measuring their pulse) and were not informed about the exact duration of the counting intervals to avoid heart beat estimations based on general knowledge. They were moreover instructed to sit comfortably during the task, to try to feel relaxed and to breathe regularly. An accommodation phase of 60 seconds preceded the actual cardiac perception measure to allow participants coming to rest and practicing the task. ECG-values were continuously recorded throughout the whole procedure.

The heartbeat perception score is considered as a valid index of IA. It bases on the comparison of the verbally reported with the ECG-recorded number of heartbeats and is calculated with the following formula: 1/3 ∑ [1 – (recorded heartbeats – reported heartbeats)/recorded heartbeats] (Herbert et al. 2012; Pollatos et al. 2007; Schandry 1981). The mean IA score is calculated across the three heartbeat-counting intervals and varies between 0 and 1. A higher score represents a smaller difference between reported and recorded heart rate i.e. higher IA. The test provides good test-retest reliability (about .81; Knoll and Hodapp 1992).

#### Suggestibility

The sensory suggestibility of the participants was assessed with the Warmth Suggestibility Scale developed by Gheorghiu et al. (2003). This standardized method bases on the application of various devices or procedures to simulate warmth stimuli or modifications of thermal sensations. In the present study, a flashlight, a medical examination lamp, a magnifying glass (diameter of 8 cm) and a contact thermode were used in the so-called initiation-, intensification-, and generalization suggestion tests to operationalize the assessment of the participants’ suggestibility to the indirect sensory suggestions. The non-existence of the suggested flashlight- or lamp-induced warmth was controlled with a digital thermometer before starting the experiment. The volunteers were instructed to inform the experimenter as soon as they perceived the feigned warmth, respectively the amplification of the thermal sensation. To simulate warmth during the initiation test, it was suggested that the flashlight would approach the closed left eyelid of the participant during the stimulation period and that the light would be visible through the eyelid. In reality, the flashlight was held at a fixed distance of about 25 centimeters, thus precluding any perceivable heat stimulus. The intensification suggestion was operationalized with the lamp kept at about 50 centimeters over the dorsal side of the left hand of the volunteer and a magnifying glass moving from below the lamp towards the hand. It was implied that the lamp would release a noticeable stable heat and that the magnifying glass would focus the light of the lamp. By approaching the glass towards the hand of the participant, an intensification of the temperature of the focused warm stimulus would possibly be felt. The warmth generalization suggestion was based on an existing heat stimulus of 32°C (neutral temperature) delivered at the palm of the dominant hand via the heat contact thermode. It was indicated that due to symmetric or balancing physiological mechanisms, a similar sensation could emerge at the opposite side of the body, either in the right hand, arm, or in any other part of the right body side. The suggestibility tests were carried out in counterbalanced order. Participants 1–20 followed the test order 1 (initiation), 2 (intensification), 3 (generalization), participants 21–40 the order 2, 1, 3, and participants 41–66 the order 3, 1, 2.

The three tests were applied once in each participant and always lasted 60 seconds. Each perception of the simulated warmth (initiation and generalization suggestion) resp. warmth modification (intensification suggestion) was verbally reported at the end of the respective trial and was scored one point. The absence of a sensory reaction was scored zero. The summed total score (range: 0–3) represented the individual and main suggestibility index. The time point at which the volunteer signalized that the simulated sensation was sensed or became more intense was considered as reaction time. This further measure of suggestibility was assessed with a stopwatch during each 0–60 seconds stimulation time range. For additional quantification of suggestibility, the evaluation of the distance observed between the magnifying glass and the hand at the moment where the intensification of the stimulation became real was assessed in centimeters. After all tests, the amount of confidence in the (non-) existence of the warmth sensations, respectively of concentration reached during the respective suggestion was rated. These additional indications on the personal extent of suggestibility were valued with a four-point Likert scale ranging from 1 = *not at all* to 4 v*ery good*. A smaller reaction time, a larger distance between the stimulus and the felt sensation, as well as a greater confidence and concentration level were considered as indicators of a higher suggestibility.

#### Experimental protocol

**Figure 3:**
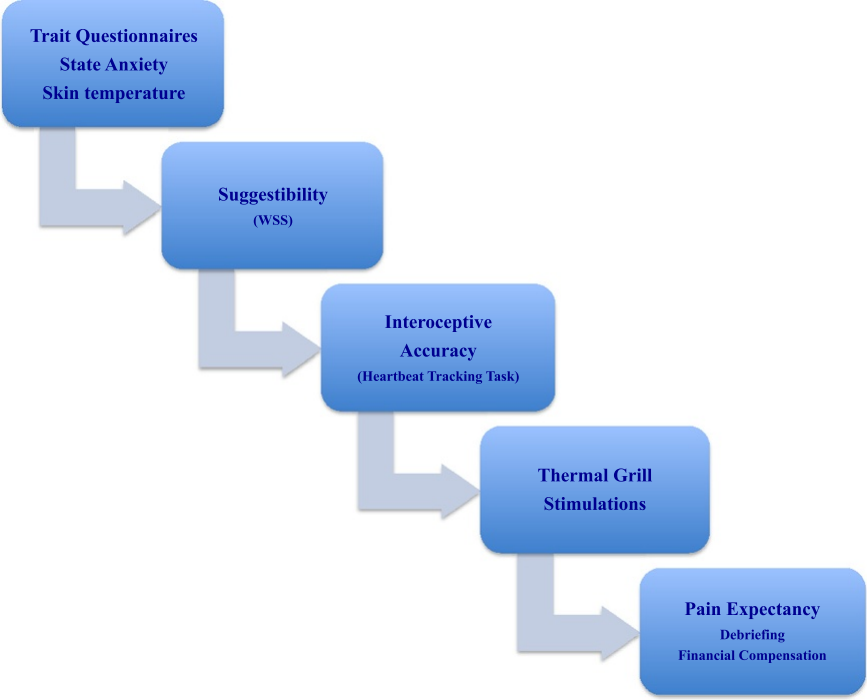
**Experimental protocol.**

The different phases of the experimental protocol are depicted in Figure 3. The same experimenter conducted all the experimental sessions (each lasting about ninety minutes) in a temperature-controlled room (22°C). The participants delivered the previously completed trait questionnaires at their arrival in the lab and filled in their responses to the STAI state anxiety items. As soon as they were seated in the test chair, the main experimental phases were described and the stimulation equipment presented. The skin temperature at the participants’ dominant hand was then measured with a digital thermometer. The experiment started with the assessment of the level of sensory suggestibility. A detailed explanation of the procedure was given before each trial. After the suggestibility assessment and detachment of the thermode from the hand of the participant, the thermal grill-related thermoelectric recirculating chillers and the contact heat stimulator were turned off to prevent all noise that might potentially hamper the subsequent heartbeat-tracking task. The ECG-electrodes were placed and a 10-minute baseline measure was done. Hereafter, IA was assessed with the heartbeat-tracking task during three time intervals of 25, 35, and 45 seconds. In a next step, the thermal grill temperatures were set at 15°C and 41°C for the experimental thermal grill condition and the procedure started. On the basis of the combined verbal/numerical rating scale, the participants orally rated pain intensity and pain unpleasantness induced by the thermal grill tubes. Following the detachment of the ECG-electrodes, the volunteers assessed the magnitude of pain they had expected to experience during the experiment on a VAS, then they were debriefed and received their financial compensation.

#### Statistical analyses

SPSS version 21 (IBM, Chicago/IL) was used for statistical analyses. The identification of responders and non-responders to paradoxical pain was based on mean pain intensity values. Mean scores were calculated by averaging the twelve reported pain values of each participant. Volunteers who had perceived more frequent and intense pain (Bouhassira et al. 2005) as expressed by higher mean scores were categorized as responders to the TGI. The responder/non-responder cut-off point in the present study was a ≥ 25-NRS score situated at equal distance between the 20-NRS score (GPT) and the 30-NRS score ‘*very weak pain*’. This score was chosen to allow the exclusion of highly variable near threshold ratings from the statistical analyses. It corresponds to 5/100-NRS on an ordinary 100 mm NRS and is in the range of values considered as a reliable indicator of pain by Boettger et al. (2013). Subjects with no or low painful sensations (mean pain ratings < 25-NRS) were hence identified as non- or poor-responders. The same 25-NRS-criterion was used for the identification of the pain unpleasantness responders and non-responders. For both pain dimensions, the sample was split in a responder and a non-responder group in terms of pain intensity and of pain unpleasantness.

Descriptive statistics for all psychophysical, psychological, and psychophysiological measures were performed for the responder and non-responder groups (see Table 1). Normal distributions of the data were examined with Kolmogorov-Smirnov tests. The pain ratings and the different characteristics of both groups were compared and analyzed for differences using non-parametric tests for non-normally distributed pain-rating and suggestibility values and t-tests for independent samples in trait/state measures with normal distribution (see Table 1). Potential associations between the different variables were assessed with Spearman’s resp. Pearson’s correlations. All trait/state analyses were run with normalized trait/state data. *P*- and *t*-values < .05 (two-tailed) were considered significant.

**Table 1:** **Absolute and statistical values of psychophysical, psychological and psychophysiological data**

Subjective pain ratings:	RESPONDERS		NON-RESPONDERS		***t***-tests	
Pain intensity:	***n*** = 24 (44.4%)		***n*** = 30 (55.6%)			
	Mean (SD)	Min-Max	Mean (SD)	Min-Max		
	38.6 (9.8)	25.4–63.3	14.4 (4.3)	2.5–24.6	*p* = .000^**^^1^	
**Pain unpleasantness**:	***n*** = **19** (35.2%)		***n*** = **35** (64.8%)			
	35.6 (11.1)	25–64.2	11.6 (8.2)	0–23.8	*p* = .000^**^	
**Trait/** **State Questionnaires/** **Tests:**						
	***n*** = **27**		***n*** = **27**		***t*** **(** ***df*** **)**	***p***
**Anxiety Trait**	40.1 (8.7)	26–60	39.8 (7.6)	26–55		
**Anxiety State**	33.6 (9.7)	0–47	30.8 (9.2)	0–44		
**Pain Catastrophizing**	17.8 (9.5)	2–31	16.1 (7.7)	1–30		
**Rumination**	13.1 (4.9)	3–25	10.9 (4.8)	3–20	1.9 (49)	.05^*^
**Optimism/** **Pessimism**	16.1 (2.9)	12–22	15.1 (4.3)	6–23		
**Interoceptive accuracy** **(IA)**	.75 (.2)	.07– .99	.61 (.2)	.09–.95	2.0 (49)	.05^*^
**Expectancy of pain**	56.1 (20)	0–85	59.6 (19.1)	15–100		
**Suggestibility** **(WSS):**	***n*** = **26**		***n*** = **27**			
**3 positive WSS tests:**	5 participants		2 participants			
**Positive Initiation test:**	12 participants		10 participants			
RT (sec)	47.3 (17.3)	5–60	51.9 (13.5)	11–60		
Confidence	2.9 (.8)	1–4	3.2 (.9)	1–4		
Concentration	3.4 (.7)	2–4	3.4 (.6)	2–4		
**Positive Intensification test:**	19 participants		18 participants			
RT (sec)	29.5 (22.3)	6–60	36.7 (20.6)	3–60		
Distance (cm)	27.1 (15.4)	5–45	24.1 (15.1)	5–50		
Confidence	3.5 (.5)	2–4	3.3 (.9)	1.5–4		
Concentration	3.4 (.8)	1–4	3.7 (.5)	2–4		
**Positive Generalisation test:**	10 participants		13 participants			
RT (sec)	52.9 (11.4)	16–60	50.4 (13.8)	15–60		
Confidence	3.2 (.8)	2–4	3.1 (.7)	1–4		
Concentration	3.6 (.6)	2–4	3.6 (.6)	2–4		

^1^ *p*-values < .05^*^ (two-tailed) were considered significant and values < .001^**^ (two-tailed) as highly significant.

Logistic regression (LR) was performed to determine which of the psychological factors of interest significantly increased the likelihood of an occurrence of a painful and/or unpleasant thermal grill illusion and to control for the accuracy of our responder/non-responder classification. Pain intensity and pain unpleasantness were used as categorical (dichotomous) dependent variables. The mean scores of non-responders (<25-NRS) were coded as 0 and higher pain values of responders (≥25-NRS) were coded as 1. All psychological and psychophysiological values were included in the LR as continuous independent variables resp. predictors, except for the suggestibility test scores (0 or 1), which figured as categorical predictors. The stepwise ‘Forward Likelihood Ratio’ procedure was employed to identify in groups of predictors those variables that provided the strongest predictive strength. Trait/state and suggestibility values were grouped separately and analyzed in distinct LR models. All predictors were logarithmically transformed (except for some categorical WSS predictors) and separately assessed for pain intensity and pain unpleasantness.

## Results

### Demographic and statistical characteristics

After exclusion of twelve participants of the total sample of tested volunteers, the data of a final sample of *N* = 54 participants [26 males, 28 females, M = 24.1 years (SD = 6.01), range 18–51 years] were analyzed. Mean pain intensity ratings were in line with other results described in the literature (Boettger et al. 2011, 2013; Bouhassira et al. 2005; see Table 1) and allowed classifying *n* = 24 participants into the category of responders (44.4%; 10 males, 14 females) and *n* = 30 into the category of the non-responders (55.6%; 16 males and 14 females) to the thermal grill illusion of pain (see Table 1). The categorization of pain unpleasantness ratings yielded *n* = 19 responders (35.2%; 10 males, 9 females) and *n* = 35 non-responders (64.8%; 16 males, 19 females) to unpleasantness of the grill stimuli (see Table 1). Overall, twenty-seven participants (50%) displayed paradoxical pain and/or pain unpleasantness responses. Sixteen responders (29.63%) reacted in both the sensory and the affective pain dimension. Twenty-seven volunteers did not (*n* = 8) or only poorly respond (*n* = 19). The assessment of the skin temperature of the participants’ dominant hand revealed a mean value of 32.89°C and a SD of 3.11.

When comparing responder and non-responder values with respect to the sensory and the affective pain ratings, non-parametric tests disclosed a highly significant difference between groups in both pain dimensions (*p* < .001). Post hoc comparisons showed that responders and non-responders differed importantly in rumination and in IA levels (*p* < .05). The investigation of the affective and cognitive personality trait and state data did mostly reveal higher mean scores in the responders than in the non-responders (see Table 1). The non-responders expected slightly more pain in the experiment than the responders and were somewhat more pessimistic. The analysis of the main WSS trials demonstrated that responders were more suggestible. Five responders felt the suggested warmth or increase of warmth in all three suggestibility tests, as compared to only two non-responding participants. During the generalization test of the WSS, the non-responders more often perceived the suggested warmth sensation in the contralateral body side. In general, the latter were slower in detecting the suggested heat sensation and perceived the simulated intensification stimulus at a smaller distance from the stimulation area. The suggestibility data of one participant were missing since this volunteer was familiar with the WSS. It should be stressed that the mentioned differences in pessimism, pain expectancy and suggestibility did not reach significance level (see Table 1).

### Spearman’s and Pearson’s correlations

Pain intensity and pain unpleasantness highly correlated when all participants were included in the analyses (*r* = .79, *N* = 54, *p* < .001). In the same total sample, pain intensity and pain unpleasantness were significantly connected to rumination (intensity: *r* = .28, *N* = 51, *p* < .05; unpleasantness: *r* = .36, *N* = 51, *p* < .01). Correlations were also found between rumination and trait anxiety (*r* = .52, *N* = 51, *p* < .001), rumination and pain catastrophizing (*r* = .44, *N* = 51, *p* ≤ .001) as well as between rumination and optimism/pessimism (*r* = −.37, *N* = 50, *p* < .01). IA correlated highly with trait anxiety (*r* = −.40, *N* = 51, *p* < .005), state anxiety (*r* = −.30, *N* = 51, *p* < .05) and optimism/pessimism (*r* = .48, *N* = 49, *p* < .001). Trait anxiety was most importantly associated to trait pain catastrophizing (*r* = .46, *N* = 54, *p* < .001), state anxiety (*r* = .36, *N* = 54, *p* < .01), and inversely to trait optimism/pessimism (*r* = −.59, *N* = 52, *p* < .001). In the group of the responding participants, optimism/pessimism was significantly related to IA (*r* = .43, *n* = 23, *p* < .05), and negatively to trait anxiety (*r* = −.56, *n* = 25, *p* < .005) and pain expectancy (*r* = −.45, *n* = 25, *p* < .05). Similar relationships as in the whole sample analyses were found in non-responders when considering correlations of rumination and IA with trait anxiety. The link between rumination-brooding values of the RSQ and those of the rumination dimension of the PCS reached significance in all groups (all *p* < .05).

Suggestibility was not linked to pain intensity sensations. The analyses of pain unpleasantness and suggestibility correlations in the whole sample however revealed a strong correlation with concentration in the intensification suggestion (*r* = −.28, *N* = 53, *p* < .05) and with reaction time in the generalization suggestion (*r* = .35, *N* = 53, *p* < .05). In non-responders, an important negative association between the affective pain component and reaction time in the intensification suggestion (*r* = −.40, *N* = 27, *p* < .05) could be observed.

### Logistic regressions (LR)

#### Predictors of the thermal grill illusion of pain

*Trait*/*state variables*. In the context of pain intensity, we focused in our first LR model on the potential impact of trait pessimism/optimism, trait pain catastrophizing, trait anxiety, trait rumination, pain expectancy, and IA on the likelihood that participants experienced the TGI. The statistically significant full model [*X*^2^ (2, *N* = 40) = 15.14, *p* < .005] showed that rumination and IA significantly contributed to the predictive ability of the model (all *p* < .05). The other independent variables did not add to the probability of a TGI occurrence. The model including rumination and IA explained between 31% (Cox and Snell R square) and 42% (Nagelkerke R square) of the variance in the TGI perception. 77.5% of the cases were correctly classified (i.e. 76.5% of the responders and 78.5% of the non-responders to the TGI). Rumination was the strongest predictor of paradoxical pain and presented an odds ratio of 35.86 (CI 2.33, 551.67; see Table 2). This result specifies that in case the rumination characteristic is under control in the model, ruminative persons are about 35 times more likely to perceive the illusion of pain than those who ruminate less. The odds ratio for IA was 20.19 (CI 1.80, 226.81; see Table 2), which signalizes that individuals who perceived their heartbeats more accurately had a 20 times higher probability to feel the paradoxical pain than less interoceptively accurate candidates. The second LR model we used included the suggestibility variables. No potential predictor of the TGI could be identified in this model.

**Table 2:** **Significant predictors of pain intensity and pain unpleasantness perceptions during thermal grill stimulation**

	B	S.E.	Wald	df	***p***	Odds ratio	95.0% C.I. for odds ratio	
Predictors for pain intensity:							Lower	Upper
Rumination	3.58	1.39	6.59	1	.01^*^^2^	35.86	2.33	551.67
Interoceptive Accuracy (IA)	3.01	1.23	5.93	1	.01^*^	20.19	1.80	226.81
**Interaction Terms:**								
State Anxiety x Rumination	.51	.21	5.75	1	.02^*^	1.67	1.10	2.55
Pain Expectancy x Rumination	.46	.20	5.40	1	.03^*^	1.48	1.04	2.13
Pessimism/Optimism x Rumination	1.03	.36	8.13	1	.004^**^	2.81	1.38	5.70
IA x Rumination	.53	.20	7.38	1	.007^*^	1.71	1.16	2.51
IA x Pain Expectancy x Rumination	.10	.04	6.49	1	.01^*^	1.11	1.02	1.20
**Predictors for pain unpleasantness:**								
Rumination	3.42	1.62	4.45	1	.03^*^	30.72	1.28	738.85
Suggestibility (WSS):								
Intensification Test – Concentration	-.88	.45	3.71	1	.05^*^	.42	.17	1.01

^2^ *p*-values < .05^*^ (two-tailed) were considered significant and values < .001^**^ (two-tailed) as highly significant.

*Trait*/*state* – *interaction terms*. The study of interacting trait/state predictors of the TGI outcome showed that rumination also considerably supported the paradoxical pain elicitation when interacting with state anxiety [*X*^2^ (1, *N* = 49) = 7.73, *p* < .05; .15 (Cox and Snell), .20 (Nagelkerke)], pain expectancy [*X*^2^ (1, *N* = 50) = 6.86, *p* < .05; .13 (Cox and Snell), .17 (Nagelkerke)], optimism/pessimism [*X*^2^ (2, *N* = 51) = 12.85, *p* < .005; .22 (Cox and Snell), .30 (Nagelkerke)], and IA [*X*^2^ (1, *N* = 48) = 10.93, *p* < .01; .20 (Cox and Snell), .27 (Nagelkerke)] (see Table 2). Between 63.3 and 75% of participants were correctly classified in these interaction models. Even a three-factor interaction term involving rumination, IA, and pain expectancy contributed significantly to the TGI prediction (*p* < .05). The predictive ability of this model was important [*X*^2^ (1, *N* = 48) = 8.84, *p* < .05] and explained between 17% and 22% of the variation in the pain intensity outcome. 75% of the participants (71.4 responders and 77.8% of non-responders) were correctly classified in the model. It could be seen that overall the likelihood of the appearance of the TGI was one to two times higher in those individuals with interacting personality features than in those without related characteristics (odds ratios varied between 1.11 and 2.81; see Table 2). It was further observed that trait anxiety and trait pain catastrophizing did not act on the probability of the TGI appearance. State anxiety, optimism/pessimism, and pain expectancy only had an effect on the prediction of pain when associated with perseverative thinking.

#### Predictors of pain unpleasantness perceptions

*Trait*/*state variables*. Regarding the prediction of pain unpleasantness outcomes in the present research, the inclusion of all previously described trait/state predictors in the logistic regression analyses again identified rumination as major influencing factor in the significant full model [*X*^2^ (1, *N* = 40) = 6.68, *p* < .05]. The predictor clarified between 15% (Cox and Snell) and 23% (Nagelkerke) of the dispersion in pain unpleasantness. The model allowed categorizing 75% of the volunteers in the appropriate group (i.e. 96.7% responders, 10% non-responders). Ruminators were 30 times more likely (Odds ratio of 30.72; CI 1.28, 738.85) to distinguish the repulsiveness of the thermal grill than non-ruminating individuals. Interacting trait/state variables did not have a predictive probability effect on the affective-motivational pain component.

*Suggestibility*-related LR results demonstrated that concentration assessed during the intensification suggestion significantly predicted the likelihood of pain unpleasantness perceptions induced by the grill (*p* ≤ .05). The model performed significantly well [*X*^2^ (1, *N* = 53) = 4.15, *p* < .05] and explained 7% to 10% of the variance in the dependent variable. Overall, 69.8% of the volunteers were correctly classified. The odds ratio of .42 inferior to 1 specified that less concentrated participants were more likely to report unpleasantness (see Table 2).

## Discussion

The psychophysical results of the present research are in agreement with previously described thermal grill-related pain ratings (Boettger et al. 2011, 2013; Bouhassira et al. 2005) and demonstrate that the applied temperature combination of 15°C and 41°C (26°C difference) yielded similar intensity and unpleasantness ratings of paradoxical pain. The evaluation of the pain scores and personality variables allowed classifying and characterizing responders and non-responders to the thermal grill stimulation paradigm. In this context, it should be emphasized that there is no generally accepted criterion for the discrimination of the two categories. As mentioned in the methods section, we chose a cut-off point of ≥ 25-NRS situated at equal distance between the 20-NRS score (GPT) and the 30-NRS score ‘very weak pain’. This value allowed us to exclude highly variable near threshold ratings from the statistical analyses. It corresponds to 5/100-NRS on standard 100 mm rating scales and hence is in the range of values considered as reliable indicators of pain (Boettger et al. 2013).

With regard to the inter-individual differences in TGI sensitivity, our results are to the best of our knowledge the first providing evidence that psychological factors in the form of cognitive and affective personality characteristics have an impact on the probability of the TGI occurrence. It could especially be established that individuals displaying high levels of trait rumination and interoceptive accuracy are more prone to perceive the illusive pain in response to the innocuous TG-stimulation. In addition, these novel findings may be relevant in the context of central neuropathic pain, which has been shown to share common neural mechanisms with respect to dysfunctional interactions between thermo-sensory and nociceptive processing (Craig et al. 1996, Craig 2008, Kern et al. 2008). The identification of significantly involved psychological factors may therefore be seen as an important contribution to the elucidation of central neuropathic pain processing and may in the longer term be relevant for the development of novel assessment and treatment strategies.

### Rumination and the thermal grill pain illusion

The strong role of rumination in the prediction of the pain illusion indicates that individuals characterized by perseverative and negative reflecting on their feelings or problems and by inactive problem-solving behaviour (Nolen-Hoeksema et al. 2008) are more pain sensitive in response to non-noxious stimulation and can feel pain where no pain should be felt. It may further be assumed that maladaptive coping with adverse contexts (Geers et al. 2008), negative expectancies of present and future outcomes (Goodin et al. 2013), and failures in deflecting attention from anticipated or on-going painful stimulations (Arntz et al. 1994; Crombez et al. 1998; Peters et al. 2000; Van Damme et al. 2004) make ruminators feel more distressed and anxious (Tang and Gibson 2005; Smith and Alloy 2009) and thus more susceptible to the TGI. In pain studies with suprathreshold noxious stimuli, it was suggested that the cognitive rumination feature may primarily influence pain perceptions when considered as a sub-factor of pain catastrophizing (Sullivan et al. 1995). In the present pain context however, the rumination trait did not act in combination with pain catastrophizing since its assessment on the basis of the Pain Catastrophizing Scale (PCS-R) did not reveal a meaningful impact. Instead, we uncovered the significant predictive capacity of the stand-alone rumination characteristic when assessing it with a pain-unspecific questionnaire, i.e. the RSQ (Nolen-Hoeksema and Morrow 1991). Nevertheless, both rumination measures, as well as rumination and pain catastrophizing correlated with each other.

### Interoceptive accuracy and the thermal grill pain illusion

The relationship between high interoceptive accuracy and enhanced affectivity or increased pain perceptions established in classical pain research (Pollatos et al. 2007, 2012) could interestingly also be observed in the present thermal grill investigation. It could be demonstrated that the ability to perceive bodily signals accurately increases the likelihood of the illusion of pain experience, a finding that may also be relevant in the context of neuropathic pain where dysfunctional thermo-sensory processes are commonly observed. The effect may possibly be explained by the circumstance that the cognitive processing of bodily cues is subjected to an emotional evaluation. With regard to the more intense emotions displayed by interoceptively accurate individuals (e.g. anxiety; Critchley et al. 2004; Krautwurst et al. 2014; Pollatos et al. 2007; Wiens et al. 2000), it has been stipulated that these strong feelings may interfere with the described affective appraisal so as to render the latter dysfunctional to a variable extent (Fairclough and Goodwin 2007; Garfinkel and Critchley 2013; Sütterlin et al. 2013; Wölk et al. 2013). In this sense, greater accuracy of estimate in the heartbeat-tracking task often revealed an association between negative cognitive appreciation of somatic cues and increased interoceptive sensitivity (Ehlers and Breuer 1996; Wölk et al. 2013). Similar impaired affective assessment of somatic signals was observed in patients displaying poorer cognitive-affective processing during decision-making processes and in healthy participants when analyzed in health anxiety and symptom report contexts (Krautwurst et al. 2014). Considering that misjudgments of interoceptive cues are held responsible for the reported enhanced somatic symptom experiences (Critchley et al. 2004) or over-reports of physical symptoms (Barsky and Borus 1999), it was proposed that anxiety-induced increases in interoceptive processing may not only maintain anxiety, but also pain which is considered to be an indicator of the physiological condition of the body (Craig 2002; Wiech and Tracey 2009). All these findings convincingly support the current finding that more accurate heartbeat perceivers are more probable to display intense paradoxical pain sensations.

### Interacting personality traits and the thermal grill pain illusion

Beside the influence of rumination per se, it could be shown here that the same cognitive characteristic also significantly increased the prediction of the TGI when interacting with anxiety, pain expectancy, pessimism, and IA. A relationship between rumination and anxiety or hostile expectations has already been demonstrated in scientific literature on depressive disorders (Nolen-Hoeksema 2000, Nolen-Hoeksema et al. 2008; Smith and Alloy 2009). Repetitive thoughts have been claimed not only to predict chronicity of depressive disorders, but also anxiety symptoms (Nolen-Hoeksema 2000), their amplification and maintenance (Segerstrom et al. 2000). Other research findings corroborated the link between rumination and anxiety by disclosing a mediating effect of rumination on the relationship between neuroticism and anxiety, respectively depression (Muris et al. 2005). The content of primarily negative ruminative thoughts, as well as pessimistic orientations and adverse expectations on present or upcoming events often seem to accompany persistent thinking (Smith and Alloy 2009). In pain research, anxiety, pain expectancy, and pessimism have mainly been related to pain catastrophizing and not to perseverative thinking since rumination is considered as a sub-factor of the multidimensional pain catastrophizing construct (Crombez et al. 1998; Edwards et al. 2006; Sullivan et al. 2001a, 2005). It has thus been recognized that increased anxiety (Sullivan et al. 2001b) and dispositional pessimism (Sinclair 2001) trigger hyperalgesia when these variables are concomitant to high pain catastrophizing. Other investigations on the impact of catastrophizing on pain perceptions and emotional distress in turn revealed that expectancy of pain mediated the relationship between catastrophizing and pain sensitivity in healthy participants (Sullivan et al. 2001b). It could furthermore be established that the magnitude of pain intensity and pain unpleasantness ratings depends on the intensity of pain an individual expects during noxious stimulation (Atlas and Wagner 2012; Tracey 2010). The more pain somebody anticipates, the more pain he will feel (Arntz et al. 1994). This relationship also reinforces expectation-based nocebo and placebo responses when influenced by anxiety and worry (Benedetti et al. 2007; Sawamoto et al. 2000). In classical pain research the interaction of rumination and IA has so far not been explored. Our findings may suggest that rumination-related negative cognitions of responders and the extent of IA, as a measure for the sensitivity to somatic signals and an indicator of emotional processing intensity, may partly interdepend. Perseverating negative thoughts and concomitant intense emotions may wind each other up and by this way exacerbate paradoxical pain sensitivity. The potentially facilitating effect of pain expectancy in the three-factor interaction with rumination and IA observed in the present study further supports the accuracy of a TGI prediction in individuals displaying negative evaluations of bodily signals. Taken together, our interaction results seem to imply that the induction of thermal grill-related pain sensations depend on affective characteristics like state anxiety, pain expectancies, dispositional pessimism, or interoceptive precision whilst cognitive factors like perseverative thoughts were possibly mainly involved in the maintenance of accompanying emotions, cognitions, and consequently paradoxical pain.

### Suggestibility and rumination in thermal grill-induced pain unpleasantness

The present research revealed that an individual’s level of suggestibility interestingly played a role in the probability of the occurrence of the affective component (unpleasantness) of the TGI rather than of the sensory-discriminative component (paradoxical pain intensity). This finding implies that more suggestible persons express preferentially unpleasantness-related sensations. It might be interesting to analyze the same suggestibility-pain unpleasantness relationship in neuropathic pain patients. In case of positive affirmation of the unraveled effect, this result might contribute to the understanding of pathological pain states that are independent of noxious input. The in literature described direct relationship between suggestibility and pain-related placebo- or nocebo effects (De Pascalis et al. 2002; Staats et al. 1998) should also be kept in mind in the clinical context.

It could moreover be observed that the cognitive factor rumination had a very strong predictive impact on the affective-motivational pain component related to the thermal grill stimulation. Other personality features did neither act alone nor in interaction with others on affective aspects of pain. The suggestibility and rumination results seem to point towards differential effects of psychological characteristics on thermal grill-related pain unpleasantness and intensity. Considering the scarcity of findings on the impact of suggestibility or rumination on pain unpleasantness in classical pain conditions, it may be hypothesized that negative cognitive processing in combination with enhanced suggestibility fostered adverse pain expectancies and were thus accountable for the unpleasant pain sensations in the current research. Further systematic research will be needed to elucidate these assumed relationships.

## Conclusion

We were able to confirm our hypothesis that the psychological factors rumination, interoceptive accuracy, and suggestibility are substantially involved in the individual pre-disposition to reporting painful sensations in the thermal grill paradigm. Further studies aiming at characterizing the impact of additional potentially involved psychological constructs (like emotional self-regulation) will be conducted to further the understanding of thermal grill-related illusive pain and concomitantly the elucidation of dysfunctional thermo-sensory processing as observed under conditions of neuropathic pain. In the long term, the respective sets of data may contribute to the development of novel assessment and treatment strategies.
